# Diaqua­bis(4-bromo­benzoato-κ^2^ *O*,*O*′)zinc(II)

**DOI:** 10.1107/S1600536808003759

**Published:** 2008-02-06

**Authors:** Tuncer Hökelek, Nagihan Çaylak, Hacali Necefoğlu

**Affiliations:** a Hacettepe University, Department of Physics, 06800 Beytepe, Ankara, Turkey; b Sakarya University, Faculty of Arts and Science, Department of Physics, 54187 Esentepe, Adapazarı, Turkey; c Kafkas University, Department of Chemistry, 63100 Kars, Turkey

## Abstract

The monomeric title Zn^II^ complex, [Zn(C_7_H_4_BrO_2_)_2_(H_2_O)_2_], contains two 4-bromo­benzoate (BB) ligands and two coordinated water mol­ecules around a Zn^II^ atom on a twofold rotation axis. The BB ions act as bidentate ligands, with two very dissimilar coordination distances. The sixfold coordination around the Zn^II^ may be described as highly distorted octa­hedral, with the two aqua ligands arranged *cis*. Hydrogen bonding involving the carboxyl­ate O atoms has an effect on the delocalization in the carboxyl­ate groups. In the crystal structure, inter­molecular O—H⋯O hydrogen bonds link the mol­ecules into chains parallel to the *c* axis and stacked along the *b* axis.

## Related literature

For general background, see: Antolini *et al.* (1982 ▶); Chen & Chen (2002 ▶); Amiraslanov *et al.* (1979 ▶); Hauptmann *et al.* (2000 ▶); Shnulin *et al.* (1981 ▶); Antsyshkina *et al.* (1980 ▶); Adiwidjaja *et al.* (1978 ▶); Catterick *et al.* (1974 ▶). For related literature, see: Guseinov *et al.* (1984 ▶); Clegg *et al.* (1986*a* ▶,*b* ▶, 1987 ▶); Capilla & Aranda (1979 ▶); van Niekerk *et al.* (1953 ▶); Usubaliev *et al.* (1992 ▶); Musaev *et al.* (1983 ▶); Nadzhafov *et al.* (1981 ▶); Day & Selbin (1969 ▶); Amiraslanov *et al.* (1980 ▶); Necefoğlu *et al.* (2002 ▶); Hökelek *et al.* (2008 ▶, 2007 ▶); Hökelek & Necefoğlu (1996 ▶, 2001 ▶, 2007 ▶); Greenaway *et al.* (1984 ▶).
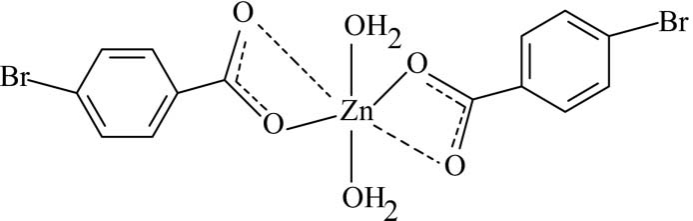


## Experimental

### 

#### Crystal data

[Zn(C_7_H_4_BrO_2_)_2_(H_2_O)_2_]
*M* *_r_* = 501.43
Monoclinic, 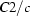

*a* = 26.9067 (3) Å
*b* = 5.0704 (4) Å
*c* = 12.0371 (5) Å
β = 104.95 (2)°
*V* = 1586.6 (2) Å^3^
*Z* = 4
Mo *K*α radiation
μ = 6.61 mm^−1^
*T* = 294 (2) K
0.25 × 0.20 × 0.15 mm

#### Data collection

Enraf–Nonius TurboCAD-4 diffractometer
Absorption correction: ψ scan (North *et al.*, 1968 ▶) *T*_min_ = 0.214, *T*_max_ = 0.370
1648 measured reflections
1613 independent reflections
1133 reflections with *I* > 2σ(*I*)
*R*_int_ = 0.031
3 standard reflections frequency: 120 min intensity decay: 1%

#### Refinement

*R*[*F* ^2^ > 2σ(*F* ^2^)] = 0.063
*wR*(*F* ^2^) = 0.166
*S* = 1.04
1613 reflections
113 parameters
4 restraints
H atoms treated by a mixture of independent and constrained refinement
Δρ_max_ = 1.42 e Å^−3^
Δρ_min_ = −1.83 e Å^−3^

### 

Data collection: *CAD-4 EXPRESS* (Enraf–Nonius, 1994 ▶); cell refinement: *CAD-4 EXPRESS*; data reduction: *XCAD4* (Harms & Wocadlo, 1995 ▶); program(s) used to solve structure: *SHELXS97* (Sheldrick, 2008 ▶); program(s) used to refine structure: *SHELXL97* (Sheldrick, 2008 ▶); molecular graphics: *ORTEP-3 for Windows* (Farrugia, 1997 ▶); software used to prepare material for publication: *WinGX* (Farrugia, 1999 ▶).

## Supplementary Material

Crystal structure: contains datablocks I, global. DOI: 10.1107/S1600536808003759/bg2161sup1.cif

Structure factors: contains datablocks I. DOI: 10.1107/S1600536808003759/bg2161Isup2.hkl

Additional supplementary materials:  crystallographic information; 3D view; checkCIF report

## Figures and Tables

**Table 1:** Selected bond lengths (Å)

Zn—O1	2.010 (5)
Zn—O2	2.468 (5)
Zn—O3	1.993 (5)

**Table 2:** Hydrogen-bond geometry (Å, °)

*D*—H⋯*A*	*D*—H	H⋯*A*	*D*⋯*A*	*D*—H⋯*A*
O3—H31⋯O2^i^	0.97 (7)	1.82 (6)	2.746 (7)	157 (9)
O3—H32⋯O1^ii^	0.95 (8)	1.86 (8)	2.765 (7)	160 (9)

Symmetry codes: (i) 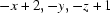; (ii) 
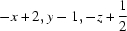
.

**Fig. 1.**
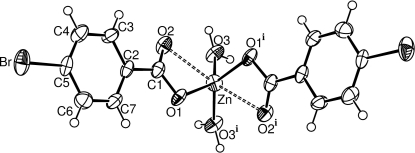
A drawing of the title molecule with the atom-numbering scheme. The displacement ellipsoids are drawn at the 50% probability level [symmetry code: (i) 2 - x, y, 1/2 - z].

## supplementary crystallographic information

### Comment

Transition metal complexes with biochemical molecules show interesting physical and/or chemical properties, through which they may find applications in biological systems (Antolini *et al.*, 1982). Some benzoic acid derivatives, such as 4-aminobenzoic acid, have been extensively reported in coordination chemistry, as bifunctional organic ligands, due to the varieties of their coordination modes (Chen & Chen, 2002, Amiraslanov *et al.*, 1979; Hauptmann *et al.*, 2000).

The structure-function-coordination relationships of the arylcarboxylate ion in Zn^II^ complexes of benzoic acid derivatives may also change depending on the nature and position of the substituted groups on the benzene ring, the nature of the additional ligand molecule or solvent, and the pH and temperature of synthesis, as in Co^II^ complexes (Shnulin *et al.*, 1981; Antsyshkina *et al.*, 1980; Adiwidjaja *et al.*, 1978). When pyridine and its derivatives are used instead of water molecules, the structure is completely different (Catterick *et al.*, 1974).

The solid-state structures of anhydrous zinc(II) carboxylates include one-dimensional (Guseinov *et al.*, 1984; Clegg *et al.*, 1986*a*), two-dimensional (Clegg *et al.*, 1986*b*, 1987) and three-dimensional (Capilla & Aranda, 1979) polymeric motifs of different types, while discerete monomeric complexes with octahedral or tetrahedral coordination geometry are found if water or other donor molecules are coordinated to Zn (van Niekerk *et al.*, 1953; Usubaliev *et al.*, 1992). In hexaaquazinc(II) bis(4-hydroxybenzoate) dihydrate, [Zn(H_2_O)_6_](4-HOC_6_H_4_COO)_2_.2H_2_O, [(II); Musaev *et al.*, 1983], which is isostructural with the corresponding Mg^II^, Co^II^, Ni^II^ and Mn^II^ compounds, the carboxylate ion lies outside the coordination sphere of the Zn atom, while [Zn(4-HOC_6_H_4_COO)_2_].4C_5_H_5_N [(III); Nadzhafov *et al.*, 1981], forms a clathrate, consisting of [Zn(4-HOC_6_H_4_COO)_2_(C_5_H_5_N)_2_] units with tetrahedral coordination geometry and free pyridine molecules.

The structure determination of the title compound, (I), a zinc complex with two bromobenzoate (BB) ligands and two water molecules, was undertaken in order to determine the ligand properties of (BB) and also to compare the results obtained with those reported previously.

In the monomeric title complex, [Zn(C_7_H_4_O_2_Br)_2_(H_2_O)_2_], (I), the Zn atom lies on a on a twofold rotation axis and is surrounded by two 4-bromobenzoate (BB), acting as bidentate ligands, and two coordinated water molecules (Fig. 1).

The Zn coordination polyhedron is formed by four clear basal bonds and two close contacts of the symmetry related O2 and O2^i^ atoms, [(i) 2 - *x*, *y*, 1/2 - *z*, Zn···O2 = 2.468 (5) Å, in double dashed lines in Fig. 1] occupying apical positions and completing the six-coordination; this distance is greater than the sum of the corresponding ionic radii (2.14 Å; Day & Selbin, 1969), but similar Zn···O contacts have already been reported, *viz*.: 2.50 (1) Å in (III), 2.494 (8) Å in [Zn(p—H_2_NC_6_H_4_COO)_2_]_n_.1.5nH_2_O [(IV); Amiraslanov *et al.*, 1980], 2.404 (2) Å in [Zn(C_6_H_6_N_2_O_2_)_2_(C_7_H_5_O_3_)_2_] [(V), (Necefoğlu *et al.*, 2002] and 2.458 (3) Å in [Zn(C_7_H_4_O_2_F)_2_(C_6_H_6_N_2_O)_2_].H_2_O [(VI); Hökelek *et al.*, 2008]. The sixfold coordination around Zn^II^ may thus be described as highly distorted octahedral (Table 1), with the two aqua ligands arranged *cis*.

In the binuclear complex [Zn_2_(C_7_H_5_O_3_)_4_(C_10_H_14_N_2_O)_2_(H_2_O)_2_] [(VII); Hökelek & Necefoğlu, 1996], the average Zn—O bond length [1.953 (2) Å] is shorter than the corresponding value in (I) [2.157 (5) Å], but Zn is four coordinate. In complexes (V), [Zn(C_7_H_4_FO_2_)_2_(DENA)_2_(H_2_O)_2_] [(VIII); Hökelek *et al.*, 2007] and [Zn(C_7_H_5_O_3_)(OH_2_)_3_(C_6_H_6_N_2_O)].C_7_O_3_H_5_ [(IX); Hökelek & Necefoğlu, 2001), (where Zn atoms are five, six and five coordinates) the average Zn—O bond lengths are 2.107 (2) Å, 2.117 (2) Å and 2.047 (5) Å, respectively. In (I), the O1—Zn···O2 angle is 57.27 (18)°. The corresponding O—M···O (where *M* is a metal) angles are 58.79 (6)° in (V), 57.04 (10)° in (VI), 58.3 (3)° in (VII) and 55.2 (1)° in [Cu(Asp)_2_(py)_2_] (where Asp is acetylsalicylate and py is pyridine) [(*X*); Greenaway *et al.*, 1984].

The near equality of C1—O1 [1.289 (8) Å] and C1—O2 [1.230 (9) Å] bonds in the carboxylate group indicates a delocalized bonding arrangement, rather than localized single and double bonds, as in (V) and [Mn(C_9_H_10_NO_2_)_2_(H_2_O)_4_].2H_2_O [(XI); Hökelek & Necefoğlu, 2007]. This may be due to the intermolecular hydrogen bonds of the carboxyl O atoms (Table 2). The Zn atom is out of the least-squares plane of the carboxyl group (O1/C1/O2) by 0.055 (1) Å. The dihedral angle between the planar carboxyl group and the benzene ring (C2–C7) is 18.62 (44)°. The corresponding value is reported as 5.54 (43)° in (XI).

The molecules of (I) are linked by intermolecular O—H···O hydrogen bonds (Table 2), forming infinite chains along the [001] direction, which are in turn stacked along the *b* axis.

### Experimental

The title compound, (I), was prepared by the reaction of ZnSO_4_ (1.61 g, 10 mmol) in H_2_O (100 ml) and *p*-bromobenzoate (4.00 g, 20 mmol) in H_2_O (100 ml). The mixture was filtered and set aside to crystallize at ambient temperature for several days, giving colorless single crystals.

### Refinement

H atoms of water molecules were located in difference syntheses and refined isotropically with restrains [O—H = 0.97 (7) and 0.95 (8) Å; *U*_iso_(H) = 0.09 (4) and 0.09 (4) Å^2^]. The remaining H atoms were positioned geometrically with C—H = 0.93 Å, for aromatic H atoms and constrained to ride on their parent atoms, with *U*_iso_(H) = 1.2*U*_eq_(C).

### Crystal data

**Table:**

[Zn(C_7_H_4_BrO_2_)_2_(H_2_O)_2_]	*F*_000_ = 976
*M**_r_* = 501.43	*D*_x_ = 2.099 Mg m^−^^3^
Monoclinic, *C*2/*c*	Mo *K*α radiation λ = 0.71073 Å
Hall symbol: -C 2yc	Cell parameters from 25 reflections
*a* = 26.9067 (3) Å	θ = 6.7–10.8º
*b* = 5.0704 (4) Å	µ = 6.61 mm^−^^1^
*c* = 12.0371 (5) Å	*T* = 294 (2) K
β = 104.95 (2)º	Block, colourless
*V* = 1586.6 (2) Å^3^	0.25 × 0.20 × 0.15 mm
*Z* = 4	

### Data collection

**Table:**

Enraf–Nonius TurboCAD-4 diffractometer	*R*_int_ = 0.031
Radiation source: fine-focus sealed tube	θ_max_ = 26.3º
Monochromator: graphite	θ_min_ = 3.1º
*T* = 294(2) K	*h* = −33→0
non–profiled ω scans	*k* = −6→0
Absorption correction: ψ scan(North *et al.*, 1968)	*l* = −14→15
*T*_min_ = 0.214, *T*_max_ = 0.370	3 standard reflections
1648 measured reflections	every 120 min
1613 independent reflections	intensity decay: 1%
1133 reflections with *I* > 2σ(*I*)	

### Refinement

**Table:**

Refinement on *F*^2^	Secondary atom site location: difference Fourier map
Least-squares matrix: full	Hydrogen site location: inferred from neighbouring sites
*R*[*F*^2^ > 2σ(*F*^2^)] = 0.063	H atoms treated by a mixture of independent and constrained refinement
*wR*(*F*^2^) = 0.166	*w* = 1/[σ^2^(*F*_o_^2^) + (0.1112*P*)^2^] where *P* = (*F*_o_^2^ + 2*F*_c_^2^)/3
*S* = 1.04	(Δ/σ)_max_ < 0.001
1613 reflections	Δρ_max_ = 1.42 e Å^−^^3^
113 parameters	Δρ_min_ = −1.83 e Å^−^^3^
4 restraints	Extinction correction: none
Primary atom site location: structure-invariant direct methods	

### Special details

**Table:**

Geometry. All e.s.d.'s (except the e.s.d. in the dihedral angle between two l.s. planes) are estimated using the full covariance matrix. The cell e.s.d.'s are taken into account individually in the estimation of e.s.d.'s in distances, angles and torsion angles; correlations between e.s.d.'s in cell parameters are only used when they are defined by crystal symmetry. An approximate (isotropic) treatment of cell e.s.d.'s is used for estimating e.s.d.'s involving l.s. planes.
Refinement. Refinement of *F*^2^ against ALL reflections. The weighted *R*-factor *wR* and goodness of fit *S* are based on *F*^2^, conventional *R*-factors *R* are based on *F*, with *F* set to zero for negative *F*^2^. The threshold expression of *F*^2^ > σ(*F*^2^) is used only for calculating *R*-factors(gt) *etc*. and is not relevant to the choice of reflections for refinement. *R*-factors based on *F*^2^ are statistically about twice as large as those based on *F*, and *R*- factors based on ALL data will be even larger.

### Fractional atomic coordinates and isotropic or equivalent isotropic displacement parameters (Å^2^)

**Table:**

	*x*	*y*	*z*	*U*_iso_*/*U*_eq_	
Br	1.22102 (3)	1.19239 (18)	0.64660 (7)	0.0502 (4)	
Zn	1.0000	0.0782 (2)	0.2500	0.0335 (4)	
O1	1.0587 (2)	0.3361 (10)	0.2873 (4)	0.0342 (12)	
O2	1.0366 (2)	0.2503 (10)	0.4458 (4)	0.0379 (12)	
O3	0.9709 (2)	−0.1998 (10)	0.3320 (4)	0.0404 (13)	
H31	0.960 (4)	−0.19 (2)	0.403 (5)	0.09 (4)*	
H32	0.953 (4)	−0.343 (15)	0.289 (7)	0.09 (4)*	
C1	1.0641 (3)	0.3712 (14)	0.3959 (6)	0.0314 (16)	
C2	1.1029 (3)	0.5711 (14)	0.4540 (5)	0.0292 (16)	
C3	1.0994 (3)	0.6756 (16)	0.5586 (6)	0.0361 (17)	
H3	1.0732	0.6214	0.5909	0.043*	
C4	1.1352 (3)	0.8615 (16)	0.6150 (6)	0.0404 (19)	
H4	1.1329	0.9337	0.6845	0.048*	
C5	1.1735 (3)	0.9357 (14)	0.5673 (6)	0.0316 (16)	
C6	1.1776 (3)	0.8399 (17)	0.4632 (7)	0.0415 (19)	
H6	1.2036	0.8983	0.4311	0.050*	
C7	1.1418 (3)	0.6525 (17)	0.4067 (6)	0.0392 (18)	
H7	1.1442	0.5826	0.3369	0.047*	

### Atomic displacement parameters (Å^2^)

**Table:**

	*U*^11^	*U*^22^	*U*^33^	*U*^12^	*U*^13^	*U*^23^
Br	0.0509 (6)	0.0389 (5)	0.0485 (5)	−0.0172 (4)	−0.0093 (4)	0.0011 (4)
Zn	0.0403 (7)	0.0166 (6)	0.0429 (7)	0.000	0.0097 (5)	0.000
O1	0.050 (3)	0.028 (3)	0.021 (2)	0.001 (2)	0.002 (2)	−0.006 (2)
O2	0.049 (3)	0.030 (3)	0.031 (3)	−0.014 (2)	0.005 (2)	0.000 (2)
O3	0.068 (4)	0.023 (3)	0.030 (3)	−0.009 (3)	0.012 (3)	−0.004 (2)
C1	0.038 (4)	0.026 (4)	0.027 (3)	0.005 (3)	0.000 (3)	−0.001 (3)
C2	0.039 (4)	0.019 (3)	0.023 (3)	0.001 (3)	−0.003 (3)	−0.003 (3)
C3	0.045 (4)	0.041 (4)	0.026 (3)	−0.012 (4)	0.014 (3)	−0.008 (3)
C4	0.054 (5)	0.035 (4)	0.030 (4)	−0.011 (4)	0.008 (3)	−0.009 (3)
C5	0.037 (4)	0.024 (3)	0.025 (3)	−0.006 (3)	−0.007 (3)	0.003 (3)
C6	0.043 (4)	0.043 (5)	0.040 (4)	−0.007 (4)	0.014 (4)	0.002 (4)
C7	0.044 (4)	0.050 (5)	0.023 (3)	−0.006 (4)	0.007 (3)	−0.009 (3)

### Geometric parameters (Å, °)

**Table:**

Br—C5	1.901 (7)	C1—C2	1.494 (10)
Zn—O1	2.010 (5)	C2—C3	1.392 (10)
Zn—O1^i^	2.010 (5)	C2—C7	1.376 (11)
Zn—O2	2.468 (5)	C3—H3	0.9300
Zn—O2^i^	2.468 (5)	C4—C3	1.393 (10)
Zn—O3	1.993 (5)	C4—H4	0.9300
Zn—O3^i^	1.993 (5)	C5—C4	1.354 (11)
O1—C1	1.289 (8)	C5—C6	1.375 (11)
O2—C1	1.230 (9)	C6—H6	0.9300
O3—H31	0.97 (7)	C7—C6	1.399 (11)
O3—H32	0.95 (8)	C7—H7	0.9300
			
O1—Zn—O1^i^	98.8 (3)	O2—C1—Zn	70.5 (4)
O1—Zn—O2	57.27 (18)	O2—C1—O1	120.1 (7)
O1^i^—Zn—O2	94.62 (19)	O2—C1—C2	123.0 (6)
O1—Zn—O2^i^	94.62 (19)	C2—C1—Zn	165.9 (5)
O1^i^—Zn—O2^i^	57.27 (18)	C3—C2—C1	118.7 (7)
O2—Zn—O2^i^	138.6 (3)	C7—C2—C1	121.6 (6)
O3^i^—Zn—O1	100.6 (2)	C7—C2—C3	119.7 (7)
O3—Zn—O1	137.4 (2)	C2—C3—C4	120.0 (7)
O3^i^—Zn—O1^i^	137.4 (2)	C2—C3—H3	120.0
O3—Zn—O1^i^	100.6 (2)	C4—C3—H3	120.0
O3^i^—Zn—O2	127.7 (2)	C3—C4—H4	120.4
O3—Zn—O2	83.58 (18)	C5—C4—C3	119.1 (7)
O3^i^—Zn—O2^i^	83.58 (18)	C5—C4—H4	120.4
O3—Zn—O2^i^	127.7 (2)	C4—C5—Br	117.6 (5)
O3^i^—Zn—O3	90.0 (3)	C4—C5—C6	122.5 (7)
C1—O1—Zn	101.1 (5)	C6—C5—Br	119.8 (6)
C1—O2—Zn	81.5 (4)	C5—C6—C7	118.3 (7)
Zn—O3—H32	119 (6)	C5—C6—H6	120.8
Zn—O3—H31	131 (6)	C7—C6—H6	120.8
H32—O3—H31	106 (4)	C2—C7—C6	120.3 (7)
O1—C1—Zn	49.6 (4)	C2—C7—H7	119.8
O1—C1—C2	116.8 (7)	C6—C7—H7	119.8
			
O1^i^—Zn—O1—C1	89.0 (4)	O1—C1—C2—C7	−19.9 (10)
O2—Zn—O1—C1	−0.8 (4)	O2—C1—C2—C7	162.8 (7)
O2^i^—Zn—O1—C1	146.6 (4)	Zn—C1—C2—C3	146.9 (18)
O3^i^—Zn—O1—C1	−129.1 (4)	Zn—C1—C2—C7	−33 (2)
O3—Zn—O1—C1	−27.4 (6)	O1—C1—C2—C3	160.3 (7)
C1^i^—Zn—O1—C1	118.5 (4)	O2—C1—C2—C3	−17.0 (11)
O1—Zn—O2—C1	0.9 (4)	C1—C2—C3—C4	179.7 (7)
O1^i^—Zn—O2—C1	−96.7 (4)	C7—C2—C3—C4	−0.1 (12)
O2^i^—Zn—O2—C1	−53.4 (4)	C1—C2—C7—C6	−180.0 (7)
O3^i^—Zn—O2—C1	78.2 (5)	C3—C2—C7—C6	−0.2 (12)
O3—Zn—O2—C1	163.1 (5)	C5—C4—C3—C2	−0.8 (12)
C1^i^—Zn—O2—C1	−78.8 (6)	Br—C5—C4—C3	179.4 (6)
Zn—O1—C1—O2	1.6 (8)	C6—C5—C4—C3	1.9 (12)
Zn—O1—C1—C2	−175.8 (5)	Br—C5—C6—C7	−179.6 (6)
Zn—O2—C1—O1	−1.3 (6)	C4—C5—C6—C7	−2.1 (12)
Zn—O2—C1—C2	175.9 (7)	C2—C7—C6—C5	1.2 (12)

Symmetry codes: (i) −*x*+2, *y*, −*z*+1/2.

### Hydrogen-bond geometry (Å, °)

**Table:**

*D*—H···*A*	*D*—H	H···*A*	*D*···*A*	*D*—H···*A*
O3—H31···O2^ii^	0.97 (7)	1.82 (6)	2.746 (7)	157 (9)
O3—H32···O1^iii^	0.95 (8)	1.86 (8)	2.765 (7)	160 (9)

Symmetry codes: (ii) −*x*+2, −*y*, −*z*+1; (iii) −*x*+2, *y*−1, −*z*+1/2.
